# Supplementary material for: Exchanging registered users’ submitting reviews towards trajectory privacy preservation for review services in Location-Based Social Networks
Source: PLoS One. 2021 Sep 16;16(9):e0256892. doi: 10.1371/journal.pone.0256892 (PMC8445480; doi:10.1371/journal.pone.0256892)
Supplement: Attachment — Submitted filename: Response to Reviewers.pdf [file pone.0256892.s002.pdf]

Original Manuscript ID: PONE-D-21-10068
Original Article Title: **“**Exchanging registered users' submitting reviews towards trajectory privacy preservation for review services in Location-Based Social Networks”

To: PLOS ONE Editor

Dear Editor,

Thank you for giving me the opportunity to submit our revised manuscript. We are uploading 4 files (a) Response to Reviewers; (b) Revised Manuscript with Track Changes; (c) Manuscript; (d) Cover letter.

Response to Reviewers contains: (a) Response to Editor; (b) Response to Reviewer#1; (c) Response to Reviewer#2.

Cover letter contains our updated Funding Statement.

Best regards,
<Yunfeng Wang> et al.

Editor, Concern # 1: Please ensure that your manuscript meets PLOS ONE's style requirements, including those for file naming. The PLOS ONE style templates can be found at.

Author response: Thank you very sincerely for giving us your comments to improve our paper. Our manuscript has been edited in accordance with the format of PLOS ONE's and we sure that our manuscript meets PLOS ONE's style requirements, including those for file naming.

Editor, Concern #2: We note that the grant information you provided in the 'Funding Information' and 'Financial Disclosure' sections do not match. When you resubmit, please ensure that you provide the correct grant numbers for the awards you received for your study in the 'Funding Information' section.

Author response: Thank you very much for your comment. We have removed the grant information in the Acknowledgments Section of our manuscript. We also re-edited our grant information and added a 'funding information updated statement' to the Cover letter.

Editor, Concern # 3: Thank you for stating the following in the Acknowledgments Section of your manuscript:"This work was partially supported by the National Key R&D Program of China under 911 Grant 2017YFB0802300, in part Major Scientific and Technological Special Project of 912 Guizhou Province under Grant 20183001,Foundation of Guizhou Provincial Key 913 Laboratory of Public Big Data under Grant 2018BDKFJJ008 and Grant 914 2018BDKFJJ021, National Statistical Scientific Research Project of China under Grant 915 2018LY61 and Grant 2019LY82, and in part Research Project of Hechi University under 916 Grant 2021KY0615 and 2021KY0620."

Author response: Thank you very much for your comment. We have removed the grant information in the Acknowledgments Section of our manuscript. We also re-edited our grant information and added a 'funding information updated statement' to the Cover letter. The updated funding information is as follows:

This research was funded by the "Major Scientific and Technological Special Project of Guizhou Province (20183001)", the "Foundation of Guizhou Provincial Key Laboratory of Public Big Data (2017BDKFJJ015, 2018BDKFJJ008, 2018BDKFJJ020, 2018BDKFJJ021)", and the "Basic Ability Improvement Program for Young and Middle-aged Teachers of Guangxi(2021KY0615 and 2021KY0620)".

Editor, Concern # 4: Please review your reference list to ensure that it is complete and correct. If you have cited papers that have been retracted, please include the rationale for doing so in the manuscript text, or remove these references and replace them with relevant current references. Any changes to the reference list should be mentioned in the rebuttal letter that accompanies your revised manuscript. If you need to cite a retracted article, indicate the article's retracted status in the References list and also include a citation and full reference for the retraction notice.

Author response: Thank you very much for your comment. According to the comment, we consulted more literatures and further improved the paper, as shown below.

~~trajectory privacy. A~~ For the other hand, a review for a ~~PoI~~ POI indicates that the owner has visited this ~~PoI. Even, some locations~~ POI. The attacker can obtain the historical trajectory of the target user by collecting (such as cyber attack [4], information sharing [5], etc.) and analyzing the historical review data of the target user. More seriously, some locations of the historical trajectory may be sensitive for

4. Vimalachandran P, Liu H, Lin YZ, JI K, Wang H, Zhang YC. Improving accessibility of the Australian My Health Records while preserving privacy and security of the system. Health Information Science and Systems. 2020; 8(1):1-9.

5. Ge YF, Yu WJ, Cao J, Wang H, Zhan ZH, et al. Distributed Memetic Algorithm for Outsourced Database Fragmentation. IEEE Transactions on Cybernetics. 2020; 99:1-14.

Evaluation Setup

Generally, privacy and utility [31,32] are two significant metrics to measure privacy pretection technology. In this section, we implement experiments on a real-world dataset to evaluate the performance of our scheme in terms of the privacy and the utility.

31. Ge YF, Cao JL, Wang H, Yin J, Yu WJ, et al. A benefit-driven genetic algorithm for balancing privacy and utility in database fragmentation. the Genetic and Evolutionary Computation Conference. 2019.

32. Ge YF, Yu WJ, Cao J, Wang H, Zhan ZH, et al. Distributed Memetic Algorithm for Outsourced Database Fragmentation. IEEE Transactions on Cybernetics. 2020; 99:1-14.

Reviewer#1, Concern # 1: Some sentences contain minor grammatical errors and need to be checked.

Author response: Thank you very much for your comment. Before we answer the following questions, we first thank you very sincerely for agreeing with my idea of our paper. According with your advice, we checked our paper carefully and modified the mistakes of grammars sentence by sentence.

Author action: We updated the manuscript by modifying the grammars of the whole paper. In our paper, since the modification is distributed in the whole paper and can't show all of them. So, please allow us to show the pictures of part of the modification. We highlighted the detailed modification in yellow

(1) The modification in the ABSTRACT.

Abstract

In Location-Based Social Networks (LBSNs), real-name registered users submit their reviews for visited point-of-interests (~~PoIs~~POIs) to the system providers (SPs). ~~These~~ The SPs anonymously publish submitted reviews to build reputations for ~~PoIs~~POIs. Unfortunately, ~~user profiles and trajectories embedded in the~~ the user profile and trajectory contained in reviews can be easily obtained by adversaries ~~compromised with SPs~~who SPs has compromised with. Even worse, existing techniques, such as cryptography and generalization, etc., are infeasible due to the necessity of ~~publishing reviews in public~~ public publication of reviews and the facticity of reviews. Inspired by pseudonym techniques, ~~this paper introduces~~ we propose an approach to exchanging reviews before users submit reviews to SPs. In our approach, we introduce two attacks, namely review-based location correlation attack (RLCA) and semantic-based long-term statistical attack (SLSA)~~, and show that exchanging reviews can be easily broken by RLCA and SLSA if we randomly exchange users ' reviews. To defend against the two attacks~~. RLCA can be exploited to link the real user by reconstructing the trajectory, and SLSA can be launched to establish a connection between locations and users through the difference of semantic frequency. To resist RLCA, we design ~~two schemes to exchange reviews that cannot be exploited to reconstruct trajectories that compromise user trajectory privacy. They can also prevent adversaries from associating an individual with consistent activities and the corresponding locations by analyzing the frequency of semantics and users in historical data.~~ a method named

(2) The modification in Section-Introduction.

Introduction

Recently, Location-Based Social Networks (LBSNs) [1] have become the dominant way people share information with others in our daily life, due to the rapid development of online social networks and the Location-Based Service. As an important component of an LBSN, Local Business Service Systems(LBSSs), such as Yelp, Tripadvisor, ~~and Dianping,~~ Dianping, etc., provide a review service [2]. In these systems, a registered user publishes a review each time she visits a point-of-interest (~~PoI~~POI) and enjoys ~~a~~ the service provided by the ~~PoI~~POI. Here, ~~every~~ a POI is a business or shop registered in an LBSS. Note that the term 'user' in this paper refers to the registered user. Users hope to build reputations for ~~these PoIs~~ POIs by publishing reviews. For example, a user ~~published~~ altruistically publishes a good review since she has enjoyed a good service of a restaurant so that more people can enjoy the service. In general, the review ~~service~~ has the following features:

(3) The modification in Section-Related work.

Related work

With growing concerns for privacy arising from prevalent LBSNs, many approaches have been proposed to protect ~~users'~~ user trajectory privacy. According to the way of

protecting trajectory privacy, these approaches can be divided into four categories: cryptography, generalization, suppression and pseudonyms.

~~In the cryptography, methods~~ Cryptography mainly encrypt and make ~~users'~~ user privacy information invisible to ~~adversaries~~ the adversary [10, 11]. However, in the ~~LBSS~~LBSSs, users' trajectories are visible for ~~adversaries~~the adversary, since the reviews are public. To overcome the vulnerability, cryptography can encrypt users' other privacy information. In [12], a user's pseudo-ID is encrypted by using a symmetric encryption algorithm. In [7], the communication between the user and the LBS server is encrypted. Nevertheless, ~~adversaries~~ the adversary can still infer users' real identities by analyzing the spatial-temporal correlation ~~of~~ between locations in trajectories [9, 13].

~~The generalization to protect a user 's trajectory privacy is mainly to hide her real~~

(4) The modification in Section-Preliminary.

Preliminary

System model and basic boncepts

~~Generally speaking, assume there is an LBSS with~~ Assume an LBSS has M POIs denoted as $\{POI_1, POI_2, \cdots, POI_M\}$ and N users denoted as $\{u_1, u_2, \cdots, u_N\}$ in a city area. ~~Although~~ Though in theory each POI POI_i has a one-to-one mapping with a unique geographic coordinate, one geographic coordinate can locate multiple POIs because of the low precision. For example, (lat:39.959679, lon:116.362065) is the FengLan International Shopping Center in Beijing, while (lat: 39.958624, lon: 116.363542) and (lat: 39.958744, lon: 116.363428) represent Watsons and Mothercare in the Shopping Center respectively. In many circumstances, some businesses or shops have the same name, such as chain shops. ~~Therefore~~So, we use a unique five-tuple to define the POI:

(5) The modification in Section- System architecture and algorithm design.

System architecture and algorithm design

System architecture

To select suitable users who exchange reviews to ~~defend against the~~ resist RLCA and SLSA, our system architecture should consider two facts: (1) ~~The~~ the SPs are adversaries and we cannot storage ~~users' historical data that has not yet been~~ non-anonymous user historical data anonymized on the LBSS server; (2) ~~The~~ the overhead of storage and calculation are huge and we cannot implement them on mobile terminals. Therefore, we ~~employ the trusted third party (TTP)~~ employcentralized architecture as our system architecture. ~~Fig 2 illustrates the system architecture . There are three main roles in our system~~Our system architecture contain three roles as shown in Fig 2.

(6) The modification in Section- Feasibility Discussion.

Feasibility Discussion

In this section, we discuss the feasibility of the proposed scheme in terms of both implementation and security. Specifically, following the aforementioned goals, we discuss whether our scheme can be implemented and achieve the desired privacy protection requirements.

Implementation analysis

Users and System Providers (SPs)

The core of our scheme is that users exchange reviews with each other. It means that for Alice, in a public review list, Bob will publish her review. Therefore, the first question we consider in our implementation analysis is whether users are willing to exchange reviews with others.

For ~~LBSNs~~LBSSs, users' identities are anonymized in various ways, such as pseudonyms~~and~~ , hiding key characters, etc. It indicates that the user is more concerned about the impact of the review on the business than about who published it. In fact, by storing the original trajectory in a database, TTP servers can still maintain authentic review lists for each user and display the review list in a way that is personally visible to each user. Therefore, it is feasible to assume that users are willing to exchange reviews to protect the trajectory privacy.

For the SPs, we mainly consider whether they are willing for users to exchange reviews with each other ~~When~~ when it is legally regulated. In general, the SPs are motivated by the desire for users to submit as many authentic reviews as possible so that they can build an objective reputation for the business. In our scenario, although users can exchange reviews, they do not submit dummy reviews as the ~~K~~ k anonymous. Therefore, it does not affect the objectivity of the ~~merchant~~business's reputation. At the same time, considering that trajectory privacy can be protected, users will be willing to submit much more reviews. So, it is feasible to assume that the SPs are willing for users to exchange reviews.

(7) The modification in Section- Feasibility Discussion.

1 ~~Evaluation Setup~~

Evaluation Setup

Generally, privacy and utility [31][32] are two significant metrics to measure privacy pretection technology. In this section, we implement experiments on a real-world dataset to evaluate the performance of our scheme in terms of the privacy and the utility.

0.1 ~~Dataset~~

Dataset

The Dataset we use for the evaluation is Yelp dataset [33] and is collected from Yelp, which is the largest review site in the United States. It contains 3 types of information: businesses, reviews and user profiles and has been used for many academic researches, such as recommendation system [34], privacy protection [2], sentiment analysis and opinion mining [35]. By pre-processing Yelp dataset, we ~~obtain~~ get a new dataset (called Combination Dataset) containing 264562 valid reviews in 510 cities for evaluating our experiments. ~~During the process, we~~ We also add a semantic field into the Combination Dataset. In general, the semantic is used to describe the ~~business's functionality~~ functionality of the business. For example, the ~~semantics~~ semantic '

(8) The modification in Section- Conclusion.

Conclusion

In this paper, we study the ~~exchange of~~ exchanging reviews for trajectory privacy protection ~~In LBSNs. The~~ in LBSSs. Since the LBSS is a registration system, adversaries can easily obtain user profiles and trajectories embedded in the reviews submitted to the SPs by compromising with the SPs~~since the LBSN is a registration system.~~ To protect trajectory privacy, we propose an approach to exchanging reviews before users ~~submit reviews to~~ submitting reviews to the SPs. However, ~~in the~~ after analysis, we find that exchanging reviews can be easily broken by RLCA and SLSA if we randomly exchange users' reviews. ~~Hence~~To resist the two attacks, we design two schemes ~~to defend the two attacks. We show that the proposed schemes can prevent~~

Reviewer#1, Concern # 2: Page 25, ''Fig 5,Fig 6,Fig 7,Fig 8'', add" and " to separate Fig 7 and Fig 8.

Author response: Thank you very much for your comment. We checked our paper carefully and modified the mistake on Page 24 and 25.

Author action: We updated the manuscript by adding the word 'and' between 'Fig 7' and 'Fig 8'. We highlighted the detailed modification in yellow as follows

(1) The modification on Page 24.

0.1 ~~Results~~

Results

(1) Privacy-preserving efficiency. We first evaluate the privacy-preserving efficiency of the USR-SLSA for our scheme. Due to the impact of different parameters on the privacy-preserving efficiency, ~~to highlight the efficiency comparison, we~~ we separately evaluate the impact of k, δ_j, δ_u, and δ_s on the privacy-preserving efficiency~~, separately~~. Fig 5, Fig 6, Fig 7, and Fig 8 show the *effective distortion ratio* for five compared algorithms with different parameters, respectively.

(2) The modification on Page 25.

Fig 5, Fig 6,Fig 7, and Fig 8 also shows some ~~of the same~~ similar evaluation results. ~~From Fig 5,Fig 6,Fig 7,Fig 8~~ Firstly we can see that the *effective distortion ratio* of USR-RLCA is larger than that of RUS. The reason is that, compared with RUS, the adversaries cannot identify more trajectories by launching RLCA when the reviews are exchanged ~~through~~ using USR-RLCA. It proves that USR-RLCA can resist RLCA. The *effective distortion ratio* of USR-SLSA is larger than that of USR-RLCA. The reason is that USR-SLSA is resistant ~~not only to RLCA but also to~~ to only both RLCA and SLSA. As a result, USR-SLSA enables fewer trajectories to be identified by the adversafy than USR-RLCA. ~~The~~ Secondly, the result of Non-exchange shows that even if users do not exchange reviews, adversaries cannot gain all users' privacy. As ~~we~~

Reviewer#1, Concern # 3: Table2 is incomplete, Please redraw it.

Author response: Thank you very much for your comment. We checked Table 2 in our paper carefully and redraw it.

Author action: We updated the manuscript by redrawing Table2. In the original manuscript, Table 2 includes 6 columns, but only 5 columns are displayed. Through modification, all columns are displayed in the revised manuscript. We highlighted the detailed modification in yellow as follows.

Table 2. Example of two trajectories exchanging reviews.

Category	Name	Category	Distorted Name	Category	
1	Public Services	6	Professional Services	11	A
2	Information Services	7	Financial Services	12	Bea
3	Home Services	8	Restaurants	13	A
4	Arts & Entertainment	9	Shopping	14	Heal
5	Life Services	10	Nightlife	15	Hot

⇨

Table 2. ~~Example~~ 15 categories of ~~two trajectories exchanging reviews~~semantics in Combination Dataset.

~~1 Public Services 6 Professional Services 11 Active Life2 Information Services 7 Financial Services 12 Beauty Spas3 Home Services 8 Restaurants 13 Automotive4 Arts Entertainment 9 Shopping 14 Health Medical5 Life Services 10 Nightlife 15 Hotels~~ ~~Travel~~

Category	Name	Category	Distorted Name	Category	Name
1	Public Services	6	Professional Services	11	Active Life
2	Information Services	7	Financial Services	12	Beauty & Spas
3	Home Services	8	Restaurants	13	Automotive
4	Arts & Entertainment	9	Shopping	14	Health & Medical
5	Life Services	10	Nightlife	15	Hotels & Travel

Reviewer#1, Concern # 4: Check the format carefully throughout the paper according to the template.

Author response: Thank you very much for your comment. We checked the format carefully

according to the template and made some modifications which are inconsistent with the format of the template. For example, delete redundant paragraphs that is not related to the manuscript, modify titles of subsections, delete the section Acknowledgment, redraw Table 2, and reedit the format of some references.

Author action: We updated the manuscript by deleting redundant paragraphs, modifying titles of subsections and deleting the section Acknowledgment, redraw Table 2, and reedit the format of some references. The detailed modifications are as follows.

(1) Delete redundant paragraphs.

Original:

Author summary

Lorem ipsum dolor sit amet, consectetur adipiscing elit. Curabitur eget porta erat. Morbi consectetur est vel gravida pretium. Suspendisse ut dui eu ante cursus gravida non sed sem. Nullam sapien tellus, commodo id velit id, eleifend volutpat quam. Phasellus mauris velit, dapibus finibus elementum vel, pulvinar non tellus. Nunc pellentesque pretium diam, quis maximus dolor faucibus id. Nunc convallis sodales ante, ut ullamcorper est egestas vitae. Nam sit amet enim ultrices, ultrices elit pulvinar, volutpat risus.

⇨ **Revised:** ~~Author summary~~

~~Lorem ipsum dolor sit amet, consectetur adipiscing elit. Curabitur eget porta erat. Morbi consectetur est vel gravida pretium. Suspendisse ut dui eu ante cursus gravida non sed sem. Nullam sapien tellus, commodo id velit id, eleifend volutpat quam. Phasellus mauris velit, dapibus finibus elementum vel, pulvinar non tellus. Nunc pellentesque pretium diam, quis maximus dolor faucibus id. Nunc convallis sodales ante, ut ullamcorper est egestas vitae. Nam sit amet enim ultrices, ultrices elit pulvinar, volutpat risus.~~

(2) Modify titles of subsections.

Original:

1 Evaluation Setup

In this section, we implement experiments on a real-world dataset to evaluate the performance of our scheme in terms of the privacy and the utility.

1.1 Dataset

The Dataset we use for the evaluation is Yelp dataset [29] and is collected from Yelp, which is the largest review site in the United States. It contains 3 types of information: businesses, reviews and user profiles and has been used for many academic researches,

⇨ **Revised:**

1 ~~Evaluation Setup~~

Evaluation Setup

Generally, privacy and utility [31,32] are two significant metrics to measure privacy pretection technology. In this section, we implement experiments on a real-world dataset to evaluate the performance of our scheme in terms of the privacy and the utility.

0.1 ~~Dataset~~

Dataset

The Dataset we use for the evaluation is Yelp dataset [33] and is collected from Yelp, which is the largest review site in the United States. It contains 3 types of information: businesses, reviews and user profiles and has been used for many academic researches,

Original:

1.2 Results

(1) Privacy-preserving efficiency. We first evaluate the privacy-preserving efficiency of the USR-SLSA for our scheme. Due to the impact of different parameters on the privacy-preserving efficiency, to highlight the efficiency comparison, we evaluate the impact of k, δ_j, δ_u, δ_s on the privacy-preserving efficiency, separately.

Fig 5, Fig 6, Fig 7, Fig 8 show the *effective distortion ratio* for five compared algorithms with different parameters, respectively.

⇨ **Revised:**

0.1 ~~Results~~

Results

(1) Privacy-preserving efficiency. We first evaluate the privacy-preserving efficiency of the USR-SLSA for our scheme. Due to the impact of different parameters on the privacy-preserving efficiency, ~~to highlight the efficiency comparison, we~~ we separately

(3) Delete the section Acknowledgment.

Original:

Acknowledgments

This work was partially supported by the National Key R&D Program of China under Grant 2017YFB0802300, in part Major Scientific and Technological Special Project of Guizhou Province under Grant 20183001,Foundation of Guizhou Provincial Key Laboratory of Public Big Data under Grant 2018BDKFJJ008 and Grant 2018BDKFJJ021, National Statistical Scientific Research Project of China under Grant 2018LY61 and Grant 2019LY82, and in part Research Project of Hechi University under Grant 2021KY0615 and 2021KY0620.

⇨ **Revised:** ~~Acknowledgments~~

~~This work was partially supported by the National Key R&D Program of China under Grant 2017YFB0802300, in part Major Scientific and Technological Special Project of Guizhou Province under Grant 20183001,Foundation of Guizhou Provincial Key Laboratory of Public Big Data under Grant 2018BDKFJJ008 and Grant 2018BDKFJJ021, National Statistical Scientific Research Project of China under Grant 2018LY61 and Grant 2019LY82, and in part Research Project of Hechi University under Grant 2021KY0615 and 2021KY0620.~~

(4) Redraw Table2.

Original:

Table 2. Example of two trajectories exchanging reviews.

Category	Name	Category	Distorted Name	Category	[illegible]
1	Public Services	6	Professional Services	11	A[illegible]
2	Information Services	7	Financial Services	12	Bea[illegible]
3	Home Services	8	Restaurants	13	A[illegible]
4	Arts & Entertainment	9	Shopping	14	Heal[illegible]
5	Life Services	10	Nightlife	15	Hot[illegible]

⇨ **Revised:**

Table 2. ~~Example~~ 15 categories of ~~two trajectories exchanging reviews~~semantics in Combination Dataset.

~~1 Public Services 6 Professional Services 11 Active Life2 Information Services 7 Financial Services 12 Beauty Spas3 Home Services 8 Restaurants 13 Automotive4 Arts Entertainment 9 Shopping 14 Health Medical5 Life Services 10 Nightlife 15 Hotels Travel~~

Category	Name	Category	Distorted Name	Category	Name
1	Public Services	6	Professional Services	11	Active Life
2	Information Services	7	Financial Services	12	Beauty & Spas
3	Home Services	8	Restaurants	13	Automotive
4	Arts & Entertainment	9	Shopping	14	Health & Medical
5	Life Services	10	Nightlife	15	Hotels & Travel

(5) Reedit the format of some references in Section-References.

References

1. Scellato S, Noulas A, Lambiotte R, Mascolo C. Socio-spatial properties of online location-based social networks. Fifth international AAAI conference on weblogs and social media, Barcelona, Spain~~, ~~.2012; 329-336~~August 2011. ~~.

2. Xu Z, Cai ZP, Li JZ, Gao H. Location-privacy-aware review publication mechanism for local business service systems. IEEE INFOCOM 2017 - IEEE Conference on Computer Communications, Atlanta, GA, USA~~, ~~. 2017~~, pp. ~~; 1-9. doi: 10.1109/INFOCOM.2017.8056976

3. Bindschaedler, V.; Shokri, R. Synthesizing Plausible Privacy-Preserving Location Traces. IEEE Symposium on Security and Privacy (S&P) IEEE, San Jose, CA, USA~~, 22–26 May 2016. ~~. 2016; 22-26. https://doi.ieeecomputersociety.org/10.1109/SP.2016.39

7. Sun YM, Chen M, Hu L, Qian YF, Hassan MM. ASA: Against statistical attacks for privacy-aware users in Location Based Service. Future generations computer systems~~: FGCS, ~~. 2017~~, ~~; 70:48-58.https://doi.org/10.1016/j.future.2016.06.017

10. Xun Y, Paulet R, Bertino E, Varadharajan V. Practical k nearest neighbor queries with location privacy. 2014 IEEE 30th International Conference on Data Engineering, Chicago, IL, USA, 2014; 640-651~~April 2014.~~.https://doi.ieeecomputersociety.org/10.1109/ICDE.2014.6816688

11. Olumofin F G, Goldberg I. Revisiting the Computational Practicality of Private Information Retrieval. Proceedings of the 15th international conference on Financial Cryptography and Data Security, Berlin, Heidelberg, ~~158 – 172 February 2011.~~2011; 158-172.https://doi.org/10.1007/978-3-642-27576-0-13

13. Xu FL, Zhen T, Yong L, Zhang PY, Fu XM, Jin DP. Trajectory Recovery From Ash: User Privacy Is NOT Preserved in Aggregated Mobility Data. Proceedings of the 26th International Conference on World Wide Web, Geneva, Switzerland, 2017; 1241-1250~~April 2017.~~.https://dl.acm.org/doi/pdf/10.1145/3038912.3052620

Reviewer#2, Concern # 1: Specific technology or method of the designed two schemes may be include in abstract.

Author response: We sincerely thank you for giving us the comment. The comment is valuable for expressing our schemes clearly. We reorganized and rewrote the sentences in Section Abstract. We also rewrote some sentences related to the comment in Section Introduction and Section Conclusion.

Author action: We updated the manuscript by reorganizing and rewriting the description related to the comment in 3 sections: Abstract, Introduction and Conclusion. The detailed modifications are as follows:

(1) Reorganize and rewrite the sentences in Section Abstract.

In our approach, we introduce two attacks, namely review-based location correlation attack (RLCA) and semantic-based long-term statistical attack (SLSA), and show that exchanging reviews can be easily broken by RLCA and SLSA if we randomly exchange users' reviews. To defend against the two attacks, we design two schemes to exchange reviews that cannot be exploited to reconstruct trajectories that compromise user trajectory privacy. They can also prevent adversaries from associating an individual with consistent activities and the corresponding locations by analyzing the frequency of semantics and users in historical data. Evaluation results verify the effectiveness of our approach in terms of privacy and utility.

⇨

to exchanging reviews before users submit reviews to SPs. In our approach, we introduce two attacks, namely review-based location correlation attack (RLCA) and semantic-based long-term statistical attack (SLSA). RLCA can be exploited to link the real user by reconstructing the trajectory, and SLSA can be launched to establish a connection between locations and users through the difference of semantic frequency. To resist RLCA, we design a method named User Selection to Resist RLCA (USR-RLCA) to exchange reviews. We propose a metric to measure the correlation between a user and a trajectory. Based on the metric, USR-RLCA can select reviews resisting RLCA to exchange by suppressing the number of locations on each reconstructed trajectory below the correlation. However, USR-RLCA fails to resist SLSA because of ignoring the essential semantics. Hence, we design an enhanced USR-RLCA named User Selection to Resist SLSA (USR-SLSA). We first propose a metric to measure the indistinguishability of locations concerning the difference of semantic frequency in a long term. Then, USR-SLSA can select reviews resisting SLSA to exchange by allowing two reviews whose indistinguishability is below the probability difference after the exchange to be exchanged. Evaluation results verify the effectiveness of our approach in terms of privacy and utility.

(2) Reorganize and rewrite the sentences in Section-Introduction.

[illegible]

- We propose two metrics. Using the first one, we design an algorithm to suppress the number of locations on each reconstructed trajectory below a threshold. Using the second one, we design an algorithm to ensure that the probability difference of semantics is below a threshold after users' reviews exchanging.

⇨

- We propose two metrics that measure the correlation between a user and a trajectory and the indistinguishability of locations concerning the difference of semantic frequency in a long term,separately. Using them, we design USR-RLCA to suppress the number of locations on each reconstructed trajectory below a threshold, and USR-SLSA to ensure that the probability difference of semantics is below a threshold after users' reviews exchanging.

(3) Reorganize and rewrite the sentences in Section- Conclusion.

exchange users' reviews. Hence, we design two schemes to defend the two attacks. We show that the proposed schemes can prevent the exchange of reviews which can be exploited to reconstruct trajectories that compromise user trajectory privacy. We also show that they can prevent adversaries from associating an individual with consistent activities and the corresponding locations. The evaluation results demonstrate that our approach can effectively protect trajectory privacy when real-name users submit their

randomly exchange users' reviews. To resist the two attacks, we design two schemes named USR-RLCA and USR-SLSA to exchange reviews. For USR-RLCA, we propose a metric to measure the correlation between a user and a trajectory. Based on the metric, USR-RLCA can select reviews resisting RLCA to exchange by suppressing the number of locations on each reconstructed trajectory below a threshold. For USR-SLSA, we propose a metric to measure the indistinguishability of locations concerning the difference of semantic frequency in a long term. Based on the metric, USR-SLSA can select reviews resisting RLCA to exchange by allowing two reviews, which the probability difference of their semantics is below a threshold after the exchange, to be exchanged. The evaluation results demonstrate that our approach can effectively

Reviewer#2, Concern # 2: What the meaning of the sentence Line63-43 Page3? It seems to be a contradiction technically feasible and revealing users' privacy.

Author response: Thank you very much for your comment. I am sorry for not clearly and accurately stating the meaning of the sentence Line63-43 Page3 in the original manuscript, due to our grammar and language issues. We rewrote these sentences and ensured that they could clearly and accurately state their meaning.

Author action: We updated the manuscript by rewriting these sentences Line63-43 Page3 in the original manuscript and ensured that these sentences could clearly and accurately state their meaning.

spatial-temporal correlation. Suppressing reviews that may reveal users' privacy is technically feasible since it does not have the disadvantages of the above two technologies. However, it reduces the utility if there are too many suppressed reviews,

⇨

spatial-temporal correlation. Suppression technique, which enables reviews that may reveal the user's privacy being invisible, is technically feasible since it does not have the disadvantages of the above two technologies. However, too many reviews being

Reviewer#2, Concern # 3: In Definition 2, the author defines a three-tuple. However, I never found the use of variable $t(r_{ij})$ again. I guess it may be used sort the set of locations in algorithms. If it's true, please adding more description in algorithms.

Author response: Thank you very much for your comment. The use of variable $t(r_{ij})$ involves two contents in the manuscript. One is used to define the trajectory T_j in Definition 2. In this definition, a trajectory T_j of u_j is a sequence of locations sorted in chronological order in which u_j has visited and reviewed the $POI(r_{ij})$. Another is used to sort the set of locations in algorithms. So, we added the description to state the use of variable $t(r_{ij})$ in Section-The algorithm design and in the Section-System model and basic concepts.

Author action: We updated the manuscript by adding the more description to state the use of variable $t(r_{ij})$. In Section-The algorithm design, we added the description to state how the set of locations are be sorted using variable $t(r_{ij})$. In Section- System model and basic concepts, we also added the description to clarify the use of variable $t(r_{ij})$ in Definition 2, namely a trajectory is a sequence of locations sorted in chronological order in which they are reviewed.

(1) Variable $t(r_{ij})$ is used to define the trajectory T_j in Definition 2 in Section-Preliminary.

Definition 2: For a user u_j, her trajectory T_j is a set of time-dependent discrete POIs reviewed in a cycle, which can be expressed as:

$$T_j = \{r_{ij} | i = 1, 2, \cdots, n\}$$

Where r_{ij} is the i-th location on T_j and denoted as a three-tuple $< POI(r_{ij}), t(r_{ij}), \tau(r_{ij}) >$, which means u_j has visited and reviewed the $POI(r_{ij})$ at time $t(r_{ij})$ in period $\tau(r_{ij})$ in a cycle. In essence, a trajectory is a sequence of locations sorted in chronological order in which they are reviewed, e.g., $t(r_{1j}) \leq t(r_{2j}) \cdots t(r_{nj})$.

(2) Variable $t(r_{ij})$ is used to sort the set of locations in algorithms in Section- System architecture and algorithm design.

1) The description in Section-RUS algorithm.

~~Secondly~~Second, u_j and other $k-1$ users are selected and their reviews are exchanged according to R. At the beginning of RUS algorithm, ~~the locations are sorted by the order of time when users visit them.~~ we first get the set of locations R' by sorting the locations in R in chronological order in which these locations are reviewed. Assume $R=\{r'_j, r'_{p_1}, r'_{p_2}, r'_{p_3}\}$. If $t(r'_{p_1}) < t(r'_{p_3}) < t(r'_{p_2})$, then $R'=\{r'_j, r'_{p_1}, r'_{p_3}, r'_{p_2}\}$. In R' ~~(the sorted set of R)~~, we first select the location r'_{pl} with the smallest time interval

---

Algorithm 1: Random-User Selection Algorithm

Input: all reviews that have not been exchanged with others, security parameter k

Output: the anonymous group G which contains k users and their reviews that have been exchanged with others, the set of the original trajectory O', the set of distorted trajectory A'.

1 Get the set of locations $R=\{r'_j, r'_{p_1}, r'_{p_2}, \cdots, r'_{p_L}\}$ from all reviews;

2 $R' \longleftarrow$ sort the locations in R ~~by the order of time~~in chronological order when the locations are reviewed;

---

2) The description in Section-RLCA algorithm.

unexchanged review to USR-RLCA algorithm. ~~The algorithm gets the set of locations~~ USR-RLCA gets the locations set $R=\{r'_j, r'_{p_1}, r'_{p_2}, \cdots, r'_{p_L}\}$ and sorts it into the ~~set of locations~~ locations set R' in ~~the order of time when users visit them~~ chronological order in which these locations are reviewed by following the method in lines 1 ~~-~~to 2 of RUS algorithm. Then, we construct the ~~set of distorted trajectories that can resist the~~

Algorithm 2: User Selection to Resist RLCA Algorithm

Input: all reviews that have not been exchanged with others, security parameter k, δ_j

Output: the anonymous group G which contains k users and their reviews that have been exchanged with others, the set of the original trajectory O', the set of distorted trajectory A'.

1 Get the set of locations $R=\{r'_j, r'_{p_1}, r'_{p_2}, \cdots, r'_{p_L}\}$ from all reviews;

2 $R' \longleftarrow$ sort the locations in R ~~by the order of time~~in chronological order when the locations are reviewed;

3 $G \longleftarrow \emptyset$, $O' \longleftarrow \emptyset$, $A' \longleftarrow \emptyset$, initial user $temp_u = u_j$,initial distorted trajectory

---

3) The description in Section-SLSA algorithm.

when receiving the reviews submitted by u_j, ~~it gives the security parameter~~ the TTP server gives k, δ_j, δ_u, δ_s to USR-SLSA algorithm. The ~~set of locations~~ locations set $R=\{r'_j, r'_{p_1}, r'_{p_2}, \cdots, r'_{p_L}\}$ should also be given and sorted ~~by the order of time (R')~~ in chronological order by following the method in lines 1 ~~-~~to 2 of USR-RLCA algorithm. Considering that we will statistic the probabilities of different users and different

---

Algorithm 3: User Selection to Resist SLSA Algorithm

Input: all reviews that have not been exchanged with others, security parameter k, δ_j, δ_u, δ_s

Output: the anonymous group G which contains k users and their reviews that have been exchanged with others, the set of the original trajectory O', the set of distorted trajectory A'.

1 Get the set of locations $R=\{r'_j, r'_{p_1}, r'_{p_2}, \cdots, r'_{p_L}\}$ from all reviews;

2 $R' \longleftarrow$ sort the locations in R ~~by the order of time~~in chronological order when the locations are reviewed;

3 Search the distorted trajectories of u_j and get the set of locations R'' which are at

---

Reviewer#2, Concern # 4: What the meaning of median in Page22 Line766? Why you only choose the data from Las Vegs?

Author response: Thank you very much for your comment. The comment pointed out the problem that we did not clearly state the reason why we only choose the data from Las Vegs. It can help to improve our paper by clearly stating the reason. In our manuscript, “median” refers to the median value. The median value is a concept in statistics and probability theory.

For a finite list of numbers, the median value is the "middle" number, when those numbers are listed in order from smallest to greatest. The advantage of the median value is that it is not skewed by a small proportion of extremely large or small values, and therefore provides a better representation of most of the numbers in the finite list.

Author action: We updated the manuscript by adding the description to state what the meaning of median is and the reason why we only choose the data from Las Vegs.

In Combination Dataset, the number of reviews varies significantly in different cities. ~~We use the~~ For example, the city with the least number of reviews only has one review, the city with the most number of reviews has thousands of reviews. It means that our evaluation is easily affected by the extreme reviews in such cities. Hence, we use the median value of the number of reviews in all cities to reduce the impact of such extreme reviews for our evaluation. The median value is a concept in statistics and probability theory. In this paper, the median value refers to the "middle" number, when the number of reviews in all cities are listed in order from smallest to greatest. But, no city has the same number of reviews as the median value. So, we use the data from Las Vegas which has the closest number of reviews to the median. The statistic for Las Vegas is shown in Table 3.

Reviewer#2, Concern # 5: In subsection Utility metric, the author points out that (ϵ, δ) is a review publication mechanism used in the literature [2] and set the value of the experiment. But I cannot find out what each of notation means.

Author response: Thank you very much for your comment. ϵ and δ are notations in literature [2]. They are thresholds and are used to balance the number of anonymous reviews and the number of public reviews for each business. In the revised manuscript, we added the description in Section- Utility metric to clarify: (1) the means of ϵ and δ are; (2) how ϵ and δ are used to preserve user location privacy; (3) the reason why we use them to publish reviews.

Author action: We updated the manuscript by adding the description in Section- Utility metric to state (1) what ϵ and δ means; (2) how ϵ and δ are used to preserve user location privacy; (3) why we use (ϵ,δ)-public principle to publish reviews.

(1) What ϵ and δ means.

Utility metric

Because users submit reviews to SP primarily for publishing, we must consider the user utility of users in terms of publication. Paper [2] is the first study on review publishing considering system utility, personal profile, and privacy in multiple regions and can preserve user location privacy by suppressing some public reviews. Since our scheme does not focus on how to publish reviews, we use ~~$(\epsilon,\delta)-publicprinciple$ to publish reviews. $(\epsilon,\delta)-publicprinciple$~~ (ϵ, δ)-public principle, which is a review publication mechanism used in the literature [2]~~. The mechanism selects reviews from users' reviews that meet $(\epsilon,\delta)-publicprinciple$ to publish~~ , to publish reviews. ϵ and δ are thresholds and are used to balance the number of anonymous reviews and the number of public reviews for each business. In the mechanism, all reviews are public when the number of the reviews for L_i (L_i refers to a business.) is less than δ. At least ϵ out of top-δ useful reviews are public when the number of the reviews for L_i is no less than δ. The mechanism can preserve users' location privacy by suppressing some public reviews. ~~This paper selects reviews from all reviews while our scheme selects~~

(2) How ϵ and δ are used to preserve user location privacy

Utility metric

Because users submit reviews to SP primarily for publishing, we must consider the user utility of users in terms of publication. Paper [2] is the first study on review publishing considering system utility, personal profile, and privacy in multiple regions and can preserve user location privacy by suppressing some public reviews. Since our scheme does not focus on how to publish reviews, we use ~~$(\epsilon,\delta) - publicprinciple$ to publish reviews. $(\epsilon,\delta) - publicprinciple$~~ (ϵ,δ)-public principle, which is a review publication mechanism used in the literature [2]~~. The mechanism selects reviews from users' reviews that meet $(\epsilon,\delta) - publicprinciple$ to publish~~, to publish reviews. ϵ and δ are thresholds and are used to balance the number of anonymous reviews and the number of public reviews for each business. In the mechanism, all reviews are public when the number of the reviews for L_i (L_i refers to a business.) is less than δ. At least ϵ out of top-δ useful reviews are public when the number of the reviews for L_i is no less than δ. The mechanism can preserve users' location privacy by suppressing some public reviews. ~~This paper selects reviews from all reviews while our scheme selects only from the reviews submitted to the LBSS server by TTP.~~

(3) Why we use (ϵ,δ)-public principle to publish reviews.

Utility metric

Because users submit reviews to SP primarily for publishing, we must consider the user utility of users in terms of publication. Paper [2] is the first study on review publishing considering system utility, personal profile, and privacy in multiple regions and can preserve user location privacy by suppressing some public reviews. Since our scheme does not focus on how to publish reviews, we use ~~$(\epsilon,\delta) - publicprinciple$ to publish reviews. $(\epsilon,\delta) - publicprinciple$~~ (ϵ,δ)-public principle, which is a review publication mechanism used in the literature [2]~~. The mechanism selects reviews from users' reviews that meet $(\epsilon,\delta) - publicprinciple$ to publish~~, to publish reviews. ϵ and δ are thresholds and are used to balance the number of anonymous reviews and the number of public reviews for each business. In the mechanism, all reviews are public when the number of the reviews for L_i (L_i refers to a business.) is less than δ. At least ϵ out of top-δ useful reviews are public when the number of the reviews for L_i is no less than δ. The mechanism can preserve users' location privacy by suppressing some public reviews. ~~This paper selects reviews from all reviews while our scheme selects only from the reviews submitted to the LBSS server by TTP.~~

Reviewer#2, Concern # 6: In Fig5, k respects number of users, the manuscript emphasizes more users in Location-Based Social Networks, but only 3 to 8 people were involved in your experiment.

Author response: Thank you very much for your comment. I am sorry for not clearly stating the meaning of the notation k. k only represents the number of users included in an anonymous group, not the number of all users in the social network. In this response, we attempt to give an example to illustrate k. Assume that there are 10 users in a social network and each user has a trajectory with 10 locations. All locations in these trajectories are different. For RLCA, we assume $\delta_j = 0.5$. For user u_j, $\delta_j = 0.5$ means that the adversary can obtain the trajectory T_u by launching the RLCA if T_u and its distorted trajectory T_u' have more than 5 locations in common after exchanging reviews. We consider that u_j only exchanges review with one user. Then we find that T_u and T_u' always have at least 5 locations in common after exchanging reviews. So u_j should exchange reviews with as many users as possible. However, more users always lead to higher overload due to the selection of more users. Therefore, we set the security parameter k to represent the number of users which exchange reviews with u_j. These users form an anonymous group. In addition, in our dataset, affected by the number of reviews of different users, the time, period, cycle, only 3-8 users can form an anonymous group. So, we added the description in Section- Results to clarify the reason why only 3 to 8 people were involved in our experiment

Author action: We updated the manuscript by adding the description in Section- Results clarify the reason why only 3 to 8 people were involved in our experiment.

Results

(1) Privacy-preserving efficiency. We first evaluate the privacy-preserving efficiency of the USR-SLSA for our scheme. Due to the impact of different parameters on the privacy-preserving efficiency, ~~to highlight the efficiency comparison, we~~ we separately evaluate the impact of k, δ_j, δ_u, and δ_s on the privacy-preserving efficiency~~, separately~~. Fig 5, Fig 6, Fig 7, and Fig 8 show the *effective distortion ratio* for five compared algorithms with different parameters, respectively.

Specifically, Fig 5 shows the change of *effective distortion ratio* when k increases. ~~From Fig 5 we can~~ Note that only reviews with the same POI, time period, etc., can be exchanged. Therefore, in our dataset, only no more than 8 users can form an anonymous group. We can see that the *effective distortion ratio* for RUS, USR-RLCA and USR-SLSA slowly reduces with the growth of k when $k < 7$ and remains constant when $k \geq 7$. This is because the number of anonymous groups that can contain at least k reviews reduces as k increases.

Reviewer#2, Concern # 7: It is noted that this paper needs more careful editing, such as grammar, and sentence structure, etc., for example: (1) Line285 Page8, T_J should be T_j; (2) Line360 Page9, $\sum_{i=2}^{m} s_i \times n_i$ should be $\sum_{i=2}^{m} n_i$; (3) Line362 Page9, s_1 should be s_i; (4) Line801 Page24, the article has no "Section 3.3", authors should notice the form of expression; (5) Line844-845 Page24, this sentence lacks the conjunction; (6) Line847 Page25, e effective e distortion ratio should be modified; (7) Line891 Page26, "In" should be "in"; (8) Table 2 is incomplete.

Author response: Thank you very much for your comment. The comment is very helpful for revising and improving our paper. We carefully checked grammar, sentence structure, etc., throughout the paper. The specific modification is as follows: (1) We made some modifications which are inconsistent with the format of the template; (2) We modified the mistakes of grammars and sentence structure sentence by sentence.

Author action: We updated the manuscript by modifying the mistakes of the format, grammars and sentence structure.

(1) Modifications involved in the comment

1)

Definition 3 (sub-trajectory): For a trajectory T_j, we assume there exists a trajectory $T' = \{r_l | l = 1, 2, \cdots, m \text{ and } m \leq n\}$, where r_l is the l-th location on T' and $\forall r_l \in T'$, ~~$r_l \in T_J$~~ $r_l \in T_j$. Let r_l and r_{l+1} be equivalent to r_{ij} and $r_{i'j}$ on T_j, respectively.

2)

Then, the number of s_1 and other semantics appearing in these POIs are $k \times n - \sum_{i=2}^{m} n_i$ and ~~$\sum_{i=2}^{m} s_i \times n_i$~~ $\sum_{i=2}^{m} n_i$, respectively. Among these $k \times n$ POIs, the frequency of s_1 is p_1, then:

3)

$k \times n$

For $\forall s_i (2 \leq i \leq m)$, the frequency of ~~$s_1$~~ s_i is p_i, then:

$$p_i = \frac{n_i}{k \times n} \quad (5)$$

4)

locations of a user as possible. For example, launching ~~the~~ SLSA to find that a location with the most frequently occurring semantic is the user's real location. Based on ~~the analysis in Section 3.3~~ Section Motivation and Basic idea, the *distortion* metric can be used to quantify the privacy. For a user and one of ~~his~~ her trajectories, if its *distortion*

5)

anonymous group. We can see that the *effective distortion ratio* for RUS, USR-RLCA and USR-SLSA slowly reduces with the growth of k when $k < 7$ and remains constant when $k \geq 7$. This is because the number of anonymous groups that can contain at least

6)

Fig 6 shows the ~~effectivedistortionratio~~ change of *effective distortion ratio* when δ_j increases. As shown in Fig 6, the *effective distortion ratio* for three algorithms hardly

7)

Conclusion

In this paper, we study the ~~exchange of~~ exchanging reviews for trajectory privacy protection ~~In LBSNs. The~~ in LBSSs. Since the LBSS is a registration system.

8)

Table 2. Example of two trajectories exchanging reviews.

Category	Name	Category	Distorted Name	Category	
1	Public Services	6	Professional Services	11	A
2	Information Services	7	Financial Services	12	Bea
3	Home Services	8	Restaurants	13	A
4	Arts & Entertainment	9	Shopping	14	Heal
5	Life Services	10	Nightlife	15	Hot

Table 2. ~~Example~~ 15 categories of ~~two trajectories exchanging reviews~~semantics in Combination Dataset.

~~1 Public Services 6 Professional Services 11 Active Life2 Information Services 7 Financial Services 12 Beauty Spas3 Home Services 8 Restaurants 13 Automotive4 Arts Entertainment 9 Shopping 14 Health Medical5 Life Services 10 Nightlife 15 Hotels Travel~~

Category	Name	Category	Distorted Name	Category	Name
1	Public Services	6	Professional Services	11	Active Life
2	Information Services	7	Financial Services	12	Beauty & Spas
3	Home Services	8	Restaurants	13	Automotive
4	Arts & Entertainment	9	Shopping	14	Health & Medical
5	Life Services	10	Nightlife	15	Hotels & Travel

(2) Made some modifications which are inconsistent with the format of the template

1) Delete redundant paragraphs.

Author summary

Lorem ipsum dolor sit amet, consectetur adipiscing elit. Curabitur eget porta erat. Morbi consectetur est vel gravida pretium. Suspendisse ut dui eu ante cursus gravida non sed sem. Nullam sapien tellus, commodo id velit id, eleifend volutpat quam. Phasellus mauris velit, dapibus finibus elementum vel, pulvinar non tellus. Nunc pellentesque pretium diam, quis maximus dolor faucibus id. Nunc convallis sodales ante, ut ullamcorper est egestas vitae. Nam sit amet enim ultrices, ultrices elit pulvinar, volutpat risus.

~~Author summary~~

~~Lorem ipsum dolor sit amet, consectetur adipiscing elit. Curabitur eget porta erat. Morbi consectetur est vel gravida pretium. Suspendisse ut dui eu ante cursus gravida non sed sem. Nullam sapien tellus, commodo id velit id, eleifend volutpat quam. Phasellus mauris velit, dapibus finibus elementum vel, pulvinar non tellus. Nunc pellentesque pretium diam, quis maximus dolor faucibus id. Nunc convallis sodales ante, ut ullamcorper est egestas vitae. Nam sit amet enim ultrices, ultrices elit pulvinar, volutpat risus.~~

2) Modify titles of subsections.

1 Evaluation Setup

In this section, we implement experiments on a real-world dataset to evaluate the performance of our scheme in terms of the privacy and the utility.

1.1 Dataset

The Dataset we use for the evaluation is Yelp dataset [29] and is collected from Yelp, which is the largest review site in the United States. It contains 3 types of information: businesses, reviews and user profiles and has been used for many academic researches,

1 ~~Evaluation Setup~~

Evaluation Setup

Generally, privacy and utility [31,32] are two significant metrics to measure privacy pretection technology. In this section, we implement experiments on a real-world dataset to evaluate the performance of our scheme in terms of the privacy and the utility.

0.1 ~~Dataset~~

Dataset

The Dataset we use for the evaluation is Yelp dataset [33] and is collected from Yelp, which is the largest review site in the United States. It contains 3 types of information: businesses, reviews and user profiles and has been used for many academic researches,

1.2 Results

(1) Privacy-preserving efficiency. We first evaluate the privacy-preserving efficiency of the USR-SLSA for our scheme. Due to the impact of different parameters on the privacy-preserving efficiency, to highlight the efficiency comparison, we evaluate the impact of k, δ_j, δ_u, δ_s on the privacy-preserving efficiency, separately. Fig 5,Fig 6,Fig 7,Fig 8 show the *effective distortion ratio* for five compared algorithms with different parameters, respectively.

0.1 ~~Results~~

Results

(1) Privacy-preserving efficiency. We first evaluate the privacy-preserving efficiency of the USR-SLSA for our scheme. Due to the impact of different parameters on the privacy-preserving efficiency, ~~to highlight the efficiency comparison, we~~ we separately

3) Delete the section Acknowledgment.

Acknowledgments

This work was partially supported by the National Key R&D Program of China under Grant 2017YFB0802300, in part Major Scientific and Technological Special Project of Guizhou Province under Grant 20183001,Foundation of Guizhou Provincial Key Laboratory of Public Big Data under Grant 2018BDKFJJ008 and Grant 2018BDKFJJ021, National Statistical Scientific Research Project of China under Grant 2018LY61 and Grant 2019LY82, and in part Research Project of Hechi University under Grant 2021KY0615 and 2021KY0620.

~~Acknowledgments~~

~~This work was partially supported by the National Key R&D Program of China under Grant 2017YFB0802300, in part Major Scientific and Technological Special Project of Guizhou Province under Grant 20183001,Foundation of Guizhou Provincial Key Laboratory of Public Big Data under Grant 2018BDKFJJ008 and Grant 2018BDKFJJ021, National Statistical Scientific Research Project of China under Grant 2018LY61 and Grant 2019LY82, and in part Research Project of Hechi University under Grant 2021KY0615 and 2021KY0620.~~

4) Redraw Table2.

Table 2. Example of two trajectories exchanging reviews.

Category	Name	Category	Distorted Name	Category	
1	Public Services	6	Professional Services	11	A
2	Information Services	7	Financial Services	12	Bea
3	Home Services	8	Restaurants	13	A
4	Arts & Entertainment	9	Shopping	14	Heal
5	Life Services	10	Nightlife	15	Hot

⇨

Table 2. ~~Example~~ 15 categories of ~~two trajectories exchanging reviews~~semantics in Combination Dataset.

~~1 Public Services 6 Professional Services 11 Active Life2 Information Services 7 Financial Services 12 Beauty Spas3 Home Services 8 Restaurants 13 Automotive4 Arts Entertainment 9 Shopping 14 Health Medical5 Life Services 10 Nightlife 15 Hotels Travel~~

Category	Name	Category	Distorted Name	Category	Name
1	Public Services	6	Professional Services	11	Active Life
2	Information Services	7	Financial Services	12	Beauty & Spas
3	Home Services	8	Restaurants	13	Automotive
4	Arts & Entertainment	9	Shopping	14	Health & Medical
5	Life Services	10	Nightlife	15	Hotels & Travel

5) Reedit the format of some references in Section-References.

References

1. Scellato S, Noulas A, Lambiotte R, Mascolo C. Socio-spatial properties of online location-based social networks. Fifth international AAAI conference on weblogs and social media, Barcelona, Spain~~,~~ .2012; 329-336~~August 2011.~~ .

2. Xu Z, Cai ZP, Li JZ, Gao H. Location-privacy-aware review publication mechanism for local business service systems. IEEE INFOCOM 2017 - IEEE Conference on Computer Communications, Atlanta, GA, USA~~,~~. 2017~~, pp.~~; 1-9. doi: 10.1109/INFOCOM.2017.8056976

3. Bindschaedler, V.; Shokri, R. Synthesizing Plausible Privacy-Preserving Location Traces. IEEE Symposium on Security and Privacy (S&P) IEEE, San Jose, CA, USA~~, 22–26 May 2016.~~. 2016; 22-26. https://doi.ieeecomputersociety.org/10.1109/SP.2016.39

7. Sun YM, Chen M, Hu L, Qian YF, Hassan MM. ASA: Against statistical attacks for privacy-aware users in Location Based Service. Future generations computer systems~~: FGCS,~~. 2017~~,~~; 70:48-58.https://doi.org/10.1016/j.future.2016.06.017

10. Xun Y, Paulet R, Bertino E, Varadharajan V. Practical k nearest neighbor queries with location privacy. 2014 IEEE 30th International Conference on Data Engineering, Chicago, IL, USA, 2014; 640-651~~April 2014.~~.https://doi.ieeecomputersociety.org/10.1109/ICDE.2014.6816688

11. Olumofin F G, Goldberg I. Revisiting the Computational Practicality of Private Information Retrieval. Proceedings of the 15th international conference on Financial Cryptography and Data Security, Berlin, Heidelberg, ~~158—172 February 2011.~~2011; 158-172.https://doi.org/10.1007/978-3-642-27576-0-13

13. Xu FL, Zhen T, Yong L, Zhang PY, Fu XM, Jin DP. Trajectory Recovery From Ash: User Privacy Is NOT Preserved in Aggregated Mobility Data. Proceedings of the 26th International Conference on World Wide Web, Geneva, Switzerland, 2017; 1241-1250~~April 2017.~~.https://dl.acm.org/doi/pdf/10.1145/3038912.3052620

(3) Made some modifications of grammars and sentence structure.

1) The modification in the ABSTRACT.

Abstract

In Location-Based Social Networks (LBSNs), real-name registered users submit their reviews for visited point-of-interests (~~PoIs~~POIs) to the system providers (SPs). ~~These~~ The SPs anonymously publish submitted reviews to build reputations for ~~PoIs~~POIs. Unfortunately, ~~user profiles and trajectories embedded in the~~ the user profile and trajectory contained in reviews can be easily obtained by adversaries ~~compromised with SPs~~who SPs has compromised with. Even worse, existing techniques, such as cryptography and generalization, etc., are infeasible due to the necessity of ~~publishing reviews in public~~ public publication of reviews and the facticity of reviews. Inspired by pseudonym techniques, ~~this paper introduces~~ we propose an approach to exchanging reviews before users submit reviews to SPs. In our approach, we introduce two attacks, namely review-based location correlation attack (RLCA) and semantic-based long-term statistical attack (SLSA)~~, and show that exchanging reviews can be easily broken by RLCA and SLSA if we randomly exchange users ' reviews. To defend against the two attacks~~. RLCA can be exploited to link the real user by reconstructing the trajectory, and SLSA can be launched to establish a connection between locations and users through the difference of semantic frequency. To resist RLCA, we design ~~two schemes to exchange reviews that cannot be exploited to reconstruct trajectories that compromise user trajectory privacy. They can also prevent adversaries from associating an individual with consistent activities and the corresponding locations by analyzing the frequency of semantics and users in historical data.~~ a method named

2) The modification in Section-Introduction.

Introduction

Recently, Location-Based Social Networks (LBSNs) [1] have become the dominant way people share information with others in our daily life, due to the rapid development of online social networks and the Location-Based Service. As an important component of an LBSN, Local Business Service Systems(LBSSs), such as Yelp, Tripadvisor, ~~and Dianping,~~ Dianping, etc., provide a review service [2]. In these systems, a registered user publishes a review each time she visits a point-of-interest (~~PoI~~POI) and enjoys ~~a~~ the service provided by the ~~PoI~~POI. Here, ~~every~~ a POI is a business or shop registered in an LBSS. Note that the term 'user' in this paper refers to the registered user. Users hope to build reputations for ~~these PoIs~~ POIs by publishing reviews. For example, a user ~~published~~ altruistically publishes a good review since she has enjoyed a good service of a restaurant so that more people can enjoy the service. In general, the review ~~service~~ has the following features:

3) The modification in Section-Related work.

Related work

With growing concerns for privacy arising from prevalent LBSNs, many approaches have been proposed to protect ~~users'~~ user trajectory privacy. According to the way of

5/33

protecting trajectory privacy, these approaches can be divided into four categories: cryptography, generalization, suppression and pseudonyms.

~~In the cryptography, methods~~ Cryptography mainly encrypt and make ~~users'~~ user privacy information invisible to ~~adversaries~~ the adversary [10, 11]. However, in the ~~LBSS~~LBSSs, users' trajectories are visible for ~~adversaries~~the adversary, since the reviews are public. To overcome the vulnerability, cryptography can encrypt users' other privacy information. In [12], a user's pseudo-ID is encrypted by using a symmetric encryption algorithm. In [7], the communication between the user and the LBS server is encrypted. Nevertheless, ~~adversaries~~ the adversary can still infer users' real identities by analyzing the spatial-temporal correlation ~~of~~ between locations in trajectories [9, 13].

~~The generalization to protect a user 's trajectory privacy is mainly to hide her real~~

4) The modification in Section-Preliminary.

Preliminary

System model and basic boncepts

~~Generally speaking, assume there is an LBSS with~~ Assume an LBSS has M POIs denoted as $\{POI_1, POI_2, \cdots, POI_M\}$ and N users denoted as $\{u_1, u_2, \cdots, u_N\}$ in a city area. ~~Although~~ Though in theory each POI POI_i has a one-to-one mapping with a unique geographic coordinate, one geographic coordinate can locate multiple POIs because of the low precision. For example, (lat:39.959679, lon:116.362065) is the FengLan International Shopping Center in Beijing, while (lat: 39.958624, lon: 116.363542) and (lat: 39.958744, lon: 116.363428) represent Watsons and Mothercare in the Shopping Center respectively. In many circumstances, some businesses or shops have the same name, such as chain shops. ~~Therefore~~So, we use a unique five-tuple to define the POI:

5) The modification in Section- System architecture and algorithm design.

System architecture and algorithm design

System architecture

To select suitable users who exchange reviews to ~~defend against the~~ resist RLCA and SLSA, our system architecture should consider two facts: (1) ~~The~~ the SPs are adversaries and we cannot storage ~~users' historical data that has not yet been~~ non-anonymous user historical data anonymized on the LBSS server; (2) ~~The~~ the overhead of storage and calculation are huge and we cannot implement them on mobile terminals. Therefore, we ~~employ the trusted third party (TTP)~~ employcentralized architecture as our system architecture. ~~Fig 2 illustrates the system architecture . There are three main roles in our system~~Our system architecture contain three roles as shown in Fig 2.

6) The modification in Section- Feasibility Discussion.

Feasibility Discussion

In this section, we discuss the feasibility of the proposed scheme in terms of both implementation and security. Specifically, following the aforementioned goals, we discuss whether our scheme can be implemented and achieve the desired privacy protection requirements.

Implementation analysis

Users and System Providers (SPs)

The core of our scheme is that users exchange reviews with each other. It means that for Alice, in a public review list, Bob will publish her review. Therefore, the first question we consider in our implementation analysis is whether users are willing to exchange reviews with others.

For ~~LBSNs~~LBSSs, users' identities are anonymized in various ways, such as pseudonyms~~and~~ , hiding key characters, etc. It indicates that the user is more concerned about the impact of the review on the business than about who published it. In fact, by storing the original trajectory in a database, TTP servers can still maintain authentic review lists for each user and display the review list in a way that is personally visible to each user. Therefore, it is feasible to assume that users are willing to exchange reviews to protect the trajectory privacy.

For the SPs, we mainly consider whether they are willing for users to exchange reviews with each other ~~When~~ when it is legally regulated. In general, the SPs are motivated by the desire for users to submit as many authentic reviews as possible so that they can build an objective reputation for the business. In our scenario, although users can exchange reviews, they do not submit dummy reviews as the ~~K~~ k anonymous. Therefore, it does not affect the objectivity of the ~~merchant~~business's reputation. At the same time, considering that trajectory privacy can be protected, users will be willing to submit much more reviews. So, it is feasible to assume that the SPs are willing for users to exchange reviews.

7) The modification in Section- Feasibility Discussion.

1 ~~Evaluation Setup~~

Evaluation Setup

Generally, privacy and utility [31][32] are two significant metrics to measure privacy pretection technology. In this section, we implement experiments on a real-world dataset to evaluate the performance of our scheme in terms of the privacy and the utility.

0.1 ~~Dataset~~

Dataset

The Dataset we use for the evaluation is Yelp dataset [33] and is collected from Yelp, which is the largest review site in the United States. It contains 3 types of information: businesses, reviews and user profiles and has been used for many academic researches, such as recommendation system [34], privacy protection [2], sentiment analysis and opinion mining [35]. By pre-processing Yelp dataset, we ~~obtain~~ get a new dataset (called Combination Dataset) containing 264562 valid reviews in 510 cities for evaluating our experiments. ~~During the process, we~~ We also add a semantic field into the Combination Dataset. In general, the semantic is used to describe the ~~business's functionality~~ functionality of the business. For example, the ~~semantics~~ semantic '

8) The modification in Section- Conclusion.

Conclusion

In this paper, we study the ~~exchange of~~ exchanging reviews for trajectory privacy protection ~~In LBSNs. The~~ in LBSSs. Since the LBSS is a registration system, adversaries can easily obtain user profiles and trajectories embedded in the reviews submitted to the SPs by compromising with the SPs~~since the LBSN is a registration system.~~. To protect trajectory privacy, we propose an approach to exchanging reviews before users ~~submit reviews to~~ submitting reviews to the SPs. However, ~~in the~~ after analysis, we find that exchanging reviews can be easily broken by RLCA and SLSA if we randomly exchange users' reviews. ~~Hence~~To resist the two attacks, we design two schemes ~~to defend the two attacks. We show that the proposed schemes can prevent~~
